# Volumetric Measurements in Lung Cancer Screening Reduces Unnecessary Low-Dose Computed Tomography Scans: Results from a Single-Center Prospective Trial on 4119 Subjects

**DOI:** 10.3390/diagnostics12020229

**Published:** 2022-01-18

**Authors:** Gianluca Milanese, Federica Sabia, Roberta Eufrasia Ledda, Stefano Sestini, Alfonso Vittorio Marchianò, Nicola Sverzellati, Ugo Pastorino

**Affiliations:** 1 Radiological Sciences, Department of Medicine and Surgery (DiMeC), University Hospital of Parma, 43126 Parma, Italy; gianluca.milanese@unipr.it (G.M.); robertaeufrasia.ledda@unipr.it (R.E.L.); nicola.sverzellati@unipr.it (N.S.); 2 Fondazione IRCCS Istituto Nazionale Tumori of Milan, 20133 Milan, Italy; federica.sabia@istitutotumori.mi.it (F.S.); stefano.sestini@istitutotumori.mi.it (S.S.); alfonso.marchiano@istitutotumori.mi.it (A.V.M.)

**Keywords:** lung cancer screening, pulmonary nodules, semi-automated volumetry

## Abstract

This study aims to compare the low-dose computed tomography (LDCT) outcome and volume-doubling time (VDT) derived from the measured volume (MV) and estimated volume (EV) of pulmonary nodules (PNs) detected in a single-center lung cancer screening trial. MV, EV and VDT were obtained for prevalent pulmonary nodules detected at the baseline round of the bioMILD trial. The LDCT outcome (based on bioMILD thresholds) and VDT categories were simulated on PN- and screenee-based analyses. A weighted Cohen’s kappa test was used to assess the agreement between diagnostic categories as per MV and EV, and 1583 screenees displayed 2715 pulmonary nodules. In the PN-based analysis, 40.1% PNs were included in different LDCT categories when measured by MV or EV. The agreements between MV and EV were moderate (κ = 0.49) and fair (κ = 0.37) for the LDCT outcome and VDT categories, respectively. In the screenee-based analysis, 46% pulmonary nodules were included in different LDCT categories when measured by MV or EV. The agreements between MV and EV were moderate (κ = 0.52) and fair (κ = 0.34) for the LDCT outcome and VDT categories, respectively. Within a simulated lung cancer screening based on a recommendation by estimated volumetry, the number of LDCTs performed for the evaluation of pulmonary nodules was higher compared with in prospective volumetric management.

## 1. Introduction

Lung cancer screening (LCS) by low-dose computed tomography (LDCT) has been proven to reduce lung cancer (LC) mortality, allowing for the early identification of pulmonary nodules (PN) with malignant behavior. Beyond traditional risk factors, size and longitudinal growth drive the management and diagnostic work-up of PNs detected on LCS [1,2,3,4]. European LCS trials support volume as a metric for assigning LDCT outcome, and the updated Lung CT screening Reporting and Data System (Lung-RADS 1.1) guidelines from the American College of Radiology (ACR) encompass volumetric thresholds for the assignment of diagnostic categories [5,6]. Although the “true” volume of a PN may be of less relevance compared with the reproducibility of measurements—particularly during longitudinal evaluations—the management of prevalent PNs depends on size, as larger PNs require shorter time intervals between LDCT rounds [7]. The scenario of inaccurate measurements of PNs less than 20 mm in diameter has been highlighted by other authors [8]. The geometrical conversion of diameters into the volume of a sphere (estimated volume, EV) is currently supported by the ACR Lung-RADS 1.1 guidelines, yet it might lead to measurement overestimation [6,9]. Conversely, measured volume (MV) of PNs obtained by dedicated software might have a positive impact on the sustainability of an LCS program by reducing the number of unnecessary LDCT rounds. However, the number of radiologists currently using dedicated software and MV in daily clinical practice is small, as most rely on diameter/EV. Nonetheless, the approaching implementation of LCS within the national health system supports the need for more accurate measurements that might be accomplished by MV.

Therefore, we report the results of a simulation performed in a single-center LCS trial, where MV is compared with EV for the assessment of both size and growth of solid PNs.

## 2. Materials and Methods

### 2.1. The bioMILD Trial

This retrospective study was performed on prospective data acquired from the baseline round of the bioMILD trial (clinicaltrials.gov ID: NCT02247453): a total of 4119 subjects were prospectively enrolled at the “Istituto Nazionale dei Tumori” of Milan (Milan, Italy) between January 2013 and March 2016. The bioMILD trial is a large prospective study testing the combination of plasma miRNA and LDCT to improve the efficacy of LCS by individual risk profiling and personalized screening intervals (clinicaltrials.gov ID: NCT02247453).

### 2.2. Imaging Acquisition

LDCT scans were performed on a second-generation dual source CT scanner (Somatom Definition Flash; Siemens Medical Solutions, Forchheim, Germany). The whole chest volume was scanned during one deep inspiratory breath-hold, with the following scanning parameters: tube voltage 120 kVp, tube current 30 mAs, collimation 0.625 mm, pitch 1.2 and rotation time 0.5 s. Images were reconstructed with the following parameters: thickness 1 mm, increment 0.7 mm, medium-sharp kernel (B50f) and lung window setting (window width 1600 HU, window level −600 HU).

### 2.3. Study Population

For the purposes of this study, we included solid PNs with a prospective software-measured maximum diameter of at least 3 mm. Diagnostic categories were assigned in keeping with the prospective thresholds of the bioMILD trial, as follows:

- Negative outcome: PN < 113 mm^3^
- Indeterminate outcome: PN 113–260 mm^3^
- Positive outcome: PN > 260 mm^3^

Analyses were performed twice: first, each PN included in the electronic database was given a diagnostic category (PN-based analysis), and then, the dominant PN was selected for each screenee (screenee-based analysis). MV was obtained by dedicated software (MM.Oncology, Syngo.via VA10; Siemens Healthcare, Forchheim, Germany). EV was calculated from measurements derived from the semi-automated analysis. Subsequently, we measured the volume doubling time (VDT) between baseline and first LDCT recall, in both the PN- and screenee-based analyses. VDTs were stratified as follows: probably malignant PN (<400 days), indeterminate PN (400–600 days), and probably benign PN (>600 days).

### 2.4. Statistical Analysis

Diameter-based nodule volume was calculated using the maximal diameter by assuming a spherical nodule shape (formula: $V=\frac{1}{6}\times \pi \times D^{3}$ with ***V*** = volume and ***D*** = maximal diameter).

VDT was calculated using the following formula: $VDT=\frac{\left[\ln2\times \Delta T\right]}{\left[\ln\left(\frac{Volume\;T2}{Volume\;T1}\right)\right]}$, where ∆***T*** is the time (in days) between the two scans.

Data were expressed as percentages. Weighted Cohen’s kappa test was used to assess the agreement between diagnostic categories as per MV and EV. All statistical analyses were performed using Statistical Analysis System software (version 9.4; SAS Institute, Cary, NC, USA).

## 3. Results

### 3.1. Study Population

In total, 1583 subjects (males: 65.5%) displayed 2715 PNs. The LDCT outcomes based on MV and EV are reported for the PN-based analysis in Table 1 and for the screenee-based analysis in Table 2.

### 3.2. PN-Based Analysis

In total, 1115 (40.1%) PNs were included in the different LDCT categories when measured by MV or EV. VDT thresholds showed that 773 PNs out of 2311 (33.4%) displayed different categories between MV-based and EV-based VDT.

Agreement between MV and EV was moderate (κ = 0.49) and fair (κ = 0.37) for the LDCT outcome and VDT categories, respectively.

### 3.3. Screenee-Based Analysis

**Figure 1:**
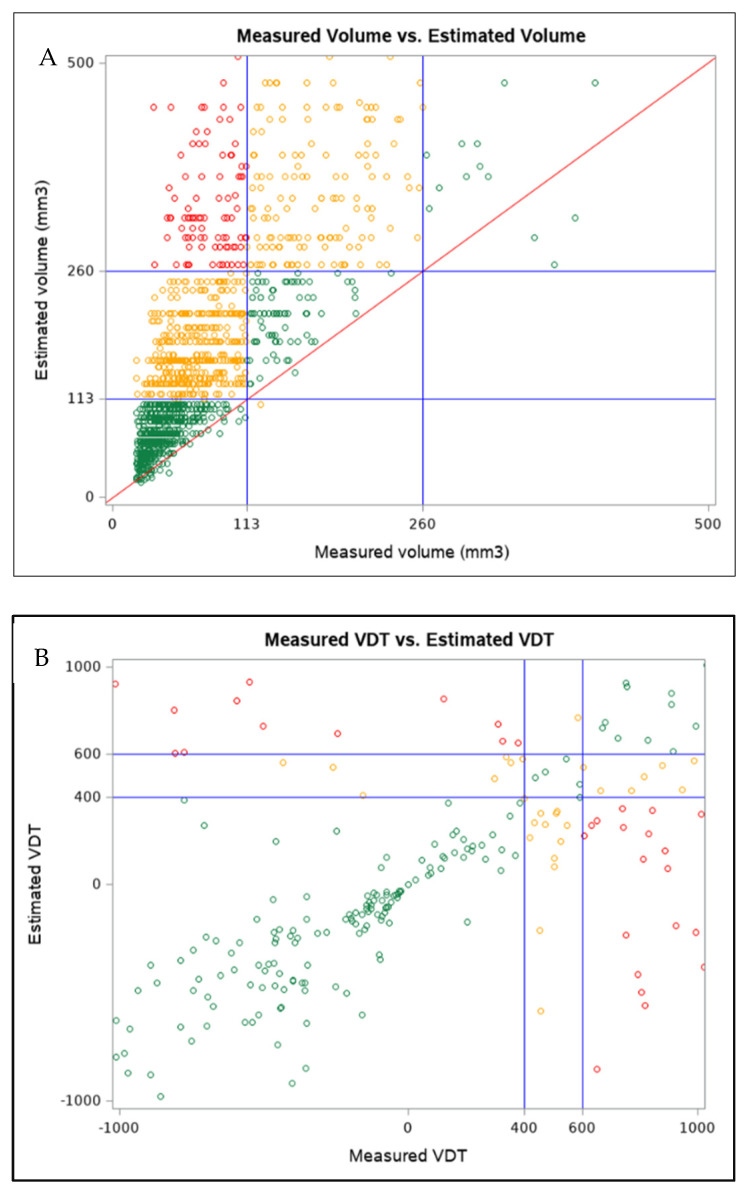
(**A**) Box plot of measured volume and estimated volume for dominant pulmonary nodules, with the volumetric thresholds for negative and positive LDCT outcome highlighted. Only dominant pulmonary nodules with volumes ranging from 3 to 500 mm^3^ are reported. (**B**) Box plot of measured volume VDT and estimated volume VDT for dominant pulmonary nodules. Only dominant pulmonary nodules with VDT within −1000 and 1000 are reported. (Color version online only).

In total, 728 (46%) PNs were included in the different LDCT categories when measured by MV or EV (Figure 1A). VDT thresholds showed that 472 dominant PNs out of 1347 (35.4%) displayed different categories between MV-based and EV-based VDT (Figure 1B).

Agreement between MV and EV was moderate (κ = 0.52) and fair (κ = 0.34) for the LDCT outcome and VDT categories, respectively.

## 4. Discussion

We compared the measured volume and the estimated volume of solid PNs detected at the baseline round of a single-center lung cancer screening trial: discrepancies between MV-based and EV-based evaluations resulted in different post-test diagnostic categories and different growth assessments, with a higher number of LDCT recalls and invasive testing from the EV-based analysis.

Volumetry of PNs has been adopted in several European LCS trials, and its measurement has been recommended for the management of PNs in daily practice by the Fleischner Society and the British Thoracic Society [10,11,12]. Indeed, MV has been recognized as more reproducible compared with diameter measurement, which suffers from limited intra- and inter-reader agreement [8]. However, beyond the definition of the most accurate method, one major question is whether one method better predicts prognosis and outcome than another [9]. Indeterminate PNs are expected in about 10–20% of screenees: in our analysis, the percentage was 16.9% for the MV-based analysis and 33.4% for the EV-based analysis. Such higher numbers of EV-based indeterminate PNs should be investigated from a clinical perspective to verify whether they include malignant lesions or are due to false-positive lesions. However, such an analysis, albeit of paramount relevance [9], goes beyond the scope of this study. We look forward to the results of the bioMILD trial, which may answer the question on personalized screening intervals based on a combination of LDCT outcome and individual risk profiling by plasma miRNA analysis.

In the PN-based analysis, 58.9% of PNs were included within the same LDCT outcome: EV caused an increased number of LDCT recalls, particularly for indeterminate PNs. The agreements between the LDCT outcome and between the VDT categories for both MV and EV were moderate and fair, respectively, with only a minor increase in the coefficient of agreement for the screenee-based analysis compared with the PN-based analysis. Risk stratification by MV might improve the affordability of LCS for national health systems, as it could grant a significant reduction in LDCT recalls throughout the LCS duration. The recently updated LungRADS categories added volumetric thresholds to diameter thresholds, and these outcome categories—assigned from semi-automated volumetric measurements of prevalent PN—could predict LC risk for screenees enrolled in the MILD trial who fulfilled the NLST selection criteria, supporting a two-year interval for negative LDCT outcome, and by reducing the number of LDCTs to be performed, such an approach might increase the sustainability of LCS [13,14]. Notably, in our screenee-based analysis, 99.9% of the differently classified PNs by EV requested a shorter follow-up compared with in the MV approach, and only three PNs were recalled following a 3-year (EV) rather than a 12-month interval (MV). The same results were derived after an evaluation of EV-based VDT, a metric that is of paramount relevance for management of PNs, either diameter-based or volumetry-based. As a variation of 20% in average diameter equates with a variation in volume of about 100%, slight differences could result in growth, requiring invasive testing for confirmation of the biological behavior of PNs [10]. This would lead to higher costs for radiology departments and, from a screenees perspective, to higher radiation exposure, higher risks from invasive maneuvers and anxiety for a greater proportion of subjects, which may cause a psychological burden that could limit engagement and adherence to LCS [5].

We recognize that PNs are not necessarily spherical and that EV—converting the maximum diameter into a sphere—intrinsically cause an overestimation of the volume of PNs. Although we did not include the mean diameter and only tested the maximum diameter obtained by a semi-automated software to limit the low reproducibility of manual measurements, our results are in keeping with those from the NELSON trial [15,16]. In daily clinical practice, however, only a minority (8%) of radiologists reported using volumetric software [17]. We aimed to test the impact of the geometrical conversion of a diameter into the volume of a sphere on the management of PNs. Such a conversion could represent an alternative to MV, particularly in settings where such tools are not available and where follow-up timing is strictly dependent on lesion measurement. Our EV approach could be considered an extension of the diameter-based approach of the NLST (e.g., LDCT-positive outcome in case of a diameter greater than a specified threshold) [1]. Furthermore, the software defines the maximum diameter with a three-dimensional approach, which may outperform the measurements obtained on axial images, which is more susceptible to a reader-based decision on slices where the maximum diameter is displayed for its manual measurement. In an anthropomorphic phantom study, greater size underestimation was reported for small PNs compared with spherical PNs [18]. Furthermore, PNs might display irregular surfaces because of transitions between nodules and the surrounding tissue (e.g., vessels or pleural surfaces). Although manual corrections of PNs’ boundaries improve the accuracy of segmentations, visual analysis of automatically segmented PNs reveals excellent results in about 80% of cases [18]. Similar values for acceptable segmentation were pursued for nodules with vascular attachments [18], nodules that—along with those that were iuxta-pleural—are known to carry challenges for accurate segmentation [19]. The potential implementation of machine-learning approaches (e.g., removal of vascular structures for accurate outlining of boundaries of PNs; radiomics analysis suggesting the risk of malignancy of PNs) might modify the management of PNs detected on LCS in the future, reducing the impact of reader intervention toward the measurement process (e.g., manual correction of inaccurate semi-automated segmentation) [18,19].

One strength of our study is the single-center setting with LDCT acquired with one single scanning protocol on a dedicated CT scanner; on the other hand, such a setting might limit generalizability of the results. Hence, we will conduct future studies comparing EV and MV in a multicentric and multivendor design. Moreover, image interpretation was performed with one software throughout the trial, allowing for a reduction in inter-scanner and inter-software variability. On the other hand, we could not test the impact of different acquisition parameters, reconstruction algorithms and software, which might influence the results of the semi-automated volumetric analysis. Our study presents some limitations. Our analyses are not stratified by nodule morphology and we excluded sub-solid PNs (about 20% of prevalent PNs was excluded), as the version of the software available at the time of the prospective reading did not allow for semi-automated volumetry analysis. We will conduct future studies testing the accuracy of newer versions of the software. As previously discussed, the results of EV might be overestimated as we considered PN as spherical structures and we did not evaluate average diameters. The results of the semi-automated segmentation were prospectively collected and information on the number of manual corrections of the PNs’ contours needed were not evaluated in the present study.

## 5. Conclusions

In conclusion, within a simulated LCS based on recommendations by estimated volumetry, the number of LDCTs performed for the evaluation of PNs is higher compared with in prospective volumetric management. Reducing the number of LDCT rounds might be beneficial for screenees—as it allows for safely reducing their overall radiation burden—as well for the national health system—as less recalls positively affect LCS sustainability.

## Figures and Tables

**Table 1:** Low-dose computed tomography (LDCT) outcomes and volume doubling time (VDT) categories derived from measured volume (MV) and estimated volume (EV) for the PN-based analysis.

		**PN-Based Analysis**		
		**EV LDCT Outcome**		
		**Negative**	**Indeterminate**	**Positive**
MV LDCT outcome	Negative	1308	735	160
	Indeterminate	3	108	217
	Positive	0	0	184
		**PN-Based Analysis**		
		**EV VDT**		
		**<400**	**400–600**	**>600**
MV VDT	<400	1021	29	254
	400–600	29	12	11
	>600	377	73	505

**Table 2:** Low-dose computed tomography (LDCT) outcomes and volume doubling time (VDT) categories derived from measured volume (MV) and estimated volume (EV) for the screenee-based analysis.

		**Screenee-Based Analysis**		
		**EV LDCT Outcome**		
		**Negative**	**Indeterminate**	**Positive**
MV LDCT outcome	Negative	611	445	100
	Indeterminate	1	83	182
	Positive	0	0	161
		**Screenee-Based Analysis**		
		**EV VDT**		
		**>600**	**400–600**	**>600**
MV VDT	<400	579	15	170
	400–600	17	5	5
	>600	225	40	291

## Data Availability

The data cannot be made publicly available due to ethical restrictions.
